# Asthma in elite athletes – do they have Type 2 or non-Type 2 disease? A new insight on the endotypes among elite athletes

**DOI:** 10.3389/falgy.2022.973004

**Published:** 2022-10-21

**Authors:** Søren Malte Rasmussen, Erik Sören Halvard Hansen, Vibeke Backer

**Affiliations:** ^1^ Medical Department, Nykøbing Falster Hospital, Nykøbing Falster, Denmark; ^2^ Centre for Physical Activity Research (CFAS), Rigshospitalet, Copenhagen, Denmark; ^3^ Department of Respiratory Medicine, Copenhagen University Hospital, Hvidovre, Hospital, Hvidovre, Denmark; ^4^ Department of Otorhinolaryngology Head / Neck surgery and Audiology, Rigshospitalet, Copenhagen University, Copenhagen, Denmark

**Keywords:** asthma, airway hyperresponsiveness (AHR), athlete, exercise, exercise-induced bronchoconstriction (EIB), asthma endotypes

## Abstract

Asthma and exercise-induced bronchoconstriction are highly prevalent in elite athletes compared with the general population. Some athletes have classic asthma with allergic sensitization; however, it seems that a proportion of athletes develop asthma as a result of several years of intensive training. It leads us to believe that asthma in athletes consists of at least two distinct endotypes – classic early-onset, Type 2 mediated asthma, and asthma with later onset caused by exercise which might be classified as non-Type 2 asthma. The purpose of this review is to evaluate the current literature on asthma in athletes focusing on inflammation and examine if asthma in athletes could be characterized as either Type 2- or non-Type 2 asthma.

## Introduction

Elite athletes are more prone to develop asthma and the prevalence of asthma among athletes are higher when compared with the general population (1). Elite training is often considered to be a contributor to the development of asthma in athletes who did not exhibit respiratory symptoms before their sports careers (2). However, not all athletes with asthma can be characterized as having the same phenotype. Therefore, it has been suggested that there are two different phenotypes of asthma in elite athletes with different endotypical characterization – athletes with classic early-onset, Type 2 mediated asthma, and athletes with asthma with later debut during their active career, which could represent another distinct asthma subtype (3). This review aimed to review the current literature to obtain information about the inflammation of asthma in athletes and to examine if athletes with asthma should be characterized with either Type 2 asthma or non-Type 2.

## Data sources and search strategy

To ensure that the literature has been reviewed three biomedical peer-review databases (PUBMED, EMBASE and Cochrane) were used in our search for published studies in a 15-year period from May 1, 2007 and until August 2, 2022. Following terms were used in the search strategy: “asthma” combined with “athlete” and “inflammation”. The search was subsequently supplemented by a manual screening of references of the selected and included papers. Searches were limited to human studies reported in English, Danish, Swedish or Norwegian. In addition, well-known studies which for unknown reasons were not found were manually included. Through database searches we identified 258 articles. The selection of studies was conducted initially by reviewing titles and abstracts, and after removing duplicates and exclusion of non-relevant studies, 63 relevant studies were reviewed.

## Asthma and exercise-induced bronchoconstriction

Asthma is established as a heterogeneous disease and asthma symptoms vary widely among patients and reflect its heterogeneity and variety of phenotypes and endotypes (4). Besides respiratory symptoms asthma is characterized by airway hyperresponsiveness (AHR) and airway inflammation. To diagnose asthma respiratory symptoms needs to be present together with examinations to objectify airway obstruction or hyperresponsiveness. The choice of assessment strategy depends on whether the patient has normal or impaired lung function. In case of impaired lung function and obstructive pattern, a reversibility test can be used, while in normal lung function an asthma provocation test must be performed. To objectify AHR in athletes bronchial provocation test is preferred. AHR can be examined by either direct- or indirect provocation test which aims to induce bronchoconstriction and observe for reduction in FEV1. Diagnostic criteria for AHR depend on the chosen test. The direct provocation tests use substances that act directly on the smooth muscle cells of the airways. Indirect tests act by affecting the inflammatory cells in the airways. The indirect tests are thus, in contrast to the direct tests, dependent on the presence of inflammation in the airways. In the diagnosis of AHR in athletes, indirect tests are recommended.

Asthma can be divided into endotypes based on the immunological conditions. Simplified, the understanding is that classic asthma is driven by T helper 2 (Th2) cells and innate lymphoid cells (ILC2), also known as Type 2 asthma, which is characterized by eosinophilic inflammation and includes both mild and severe allergic asthma as well as non-allergic eosinophilic asthma. Non-Type 2 asthma is classified by the absence of Type 2 inflammatory parameters such as blood eosinophil cells, sputum eosinophils and elevated fractional exhaled nitric oxide (FeNO) (5).

Exercise-induced bronchoconstriction (EIB) is a condition where acute airway narrowing occurs during or in a short period after exercise is ended and leads to respiratory symptoms such as shortness of breath, cough, and wheeze. EIB is often seen in patients with asthma but are also found in individuals without asthma and is especially highly present among athletes (6).

## Why does exercise trigger bronchoconstriction?

The exact pathophysiological mechanisms underlying EIB are unclear, and several factors are known triggers. In swimmers, the inhalation of chlorine has been suggested as a trigger and in winter sports cold air has been suggested. But these factors do not explain EIB in athletes in general (2). Therefore, three theories of EIB have been proposed: *The thermal theory*, *the osmotic theory*, and *the recurrent epithelial microtrauma theory* (7–9). The *thermal theory* suggests that increased ventilation induce changes in temperature which leads to dehydration of the airway walls subsequently causing local vasoconstriction. When the airway walls regain their normal temperature, the blood vessels become hyperpermeable and distend causing bronchial wall edema and subsequently bronchoconstriction (2, 10). The *osmotic theory* suggests that increased ventilation cause dehydration of the airways which leads to a hyperosmotic state and stimulates the release of inflammatory mediators which induce bronchial smooth muscle contraction (11). The *recurrent microtrauma* theory suggests that the sheer stress of increased ventilation causes injury to the airway epithelium which involves microvascular leak and plasma exudation. This repeated injury-repair process result in changes in the contractile properties of the airway smooth muscle following exposure to plasma derived products leading to development and progression in airway hyperresponsiveness in athletes (11–14).

## Prevalence of asthma and EIB in athletes

Depending on the study population and diagnostic criteria the prevalence of asthma and EIB in elite athletes are estimated to be as high as 55% (15–17). The high prevalence of asthma and EIB in athletes seems to be depending on the type of sport. Studies have repeatedly shown that the prevalence of asthma is particularly frequent among endurance disciplines such as swimming, rowing, cycling and winter sport disciplines e.g., cross-country- and biathlon skiers (18–22). A recent study on more than 1,300 European athletes from the summer Olympics found that asthma prevalence was estimated to 16.5% across all types of sport and most often were found among endurance athletes (55.7%) (23).

## What characterizes athletes with asthma?

Regarding the type of sport, athletes competing on elite levels often train two or three times per day, which results in 20–30 h of training per week. In resting state, the respiratory minute volume (VE) is between 5 and 8 L/min. The high intensity training in endurance sport requires high ventilation frequency and ventilation compared with resting state can increase with 20–30 fold (L/min) (24). This increased ventilation during training is suspected as an essential component in the development of asthma in elite athletes.

Besides high prevalence of asthma, athletes often have concurrent allergy and rhinitis and increased risk of upper respiratory tract infection (25–28). Allergic and respiratory symptoms are highly prevalent in both athletes with and without asthma and seem to be influenced by the type of sport and exercise environment (29–32). Elite athletes with asthma primarily have symptoms related to exercise, and only few athletes have symptoms during rest (33). Detailed knowledge about respiratory symptoms can be useful, as they may indicate asthma, but as exercise-induced symptoms have poor predictive value of asthma, a diagnosis of asthma in elite athletes requires documentation of AHR (18, 34). These asthma-like-symptoms might also be influenced by conditions as irritants or allergens in the external environment surrounding the athlete, e.g., swimmer's exposure to chlorine, urban and outdoor competing athletes' inhalation of traffic pollution and allergens or winter sport athletes' exposure to cold and dry air. Furthermore, in some sports there might be a combination of more than one exposure e.g., triathlon.

It is well known that asthma in general often debuts in childhood as well as early adolescence (early onset). In these age groups patients are suspected to have Th2 driven asthma. Studies have found that asthma among Olympic athletes develops after the age of 20 years, which is considered as late-onset asthma. The same study showed that only a third of these athletes had reported childhood asthma, which suggest that two thirds of elite athletes develop asthma later in their life and during their active sport career (35). Moreover, studies on skiers have found that the age of asthma onset varied from early adolescence to early adulthood (36). A study on Swedish adolescent elite skiers showed that the median age of asthma onset was higher among skiers than non-athletes (37) and other studies on elite skiers found that athletes recalled their onset age of asthma to be around late adolescence or early adulthood (38). None out of 42 skiers with asthma recalled debut of asthma to be during childhood (39). Although some athletes with asthma are diagnosed in childhood or early adolescence, studies support the hypothesis that asthma in athletes are not always present in the beginning their career (40). One thing is the presence of asthma, another is the presence of EIB in young athletes. A study on athletes between 12 and 14 years in the beginning of their elite sports career found that EIB is present in a substantial number of individuals (41). Moreover, Jonckheere et al. found that 24.5% of young athletes (12–13 years) suffer from EIB (42). However other studies on young athletes did not find increased EIB or signs of airway inflammation (40, 43). Early onset of EIB in young athletes without asthma might be considered as a risk factor for the development of asthma later in the career.

## Airway inflammation

Several studies have examined the presence and type of airway inflammation in elite athletes both with and without asthma and at rest or after exercise*.* It is suspected that differences between the subtypes of asthma in athletes might be due to different occurrence of airway inflammation. However, the exact role of airway inflammation in athletes are not fully understood and studies are showing conflicting results.

Studies on athletes competing in different disciplines have shown increased presence of neutrophilic airway inflammation (44–47). A study by Stang et al. aimed to assess the long-term change in airway inflammatory response to endurance exercise in athletes with and without asthma (48). Results found increased levels of sputum IL-8 in athletes compared with healthy non-athletes, independent of asthma diagnosis. Sue-Chu et al. (49) have shown that the airway inflammation among elite athletes (skiers) exposed to cold air is mostly represented by macrophages and neutrophils suggesting non-Type-2 inflammation, whereas classic asthma patients, characterized as early-onset childhood asthma, in general have eosinophilic infiltration (50). Moreover, number of training hours per week correlated with sputum neutrophil count in both swimmers and cold-air athletes (51). Other studies find low or no differences in airway inflammation when athletes are compared with non-athletes with asthma(40).

In contrast, other studies find increased occurrence of eosinophilic airway inflammation in EIB positive asthma patients (52). While some studies on elite athletes with asthma found a mixed type of eosinophilic, lymphocyte and neutrophilic inflammation in sputum samples, which might be related to their specific type of sport (46, 53, 54).

Besides eosinophils and neutrophils, mast cells seem to play a central role in airway inflammation in both Type 2 and non-Type 2 asthma. The link between mast cells and indirect AHR and expression of Type 2 inflammatory genes in the airways are supported by recent studies (55–58). Hallstrand and colleagues has shown that mast cells in healthy controls are located predominantly in submucosa but seem to change location in patients with asthma where they are found in the epithelial layer and might be closely associated with airway dysfunction in the form of indirect AHR (55). The same group showed correlation between mast cell location in epithelium layer and AHR together with expression of Type 2 inflammatory markers in sputum (55). A study by Al-Shaikhly et al. examined eosinophils in the different airway wall compartments (epithelial and subepithelial spaces) by endobronchial biopsy samples. Results showed that patients with T2-high asthma had higher densities of eosinophils in the airway wall overall, the subepithelial and the epithelial compartments when compared to T2-low asthma patients. Moreover, results found that intraepithelial eosinophils are a unique feature of asthma and are related to features of endogenous AHR and Type 2 inflammation. Presence of intraepithelial mast cells have been described earlier and found as a pathologic characteristic of T2-high asthma (59). Another important finding from Al-Shaikhly et al. is the interaction between eosinophils and mast cells within the airway epithelial compartment through LTC_4_-pathways (60). Paucigranulocytic asthma may also be important in athletes, and the absence of neutrophils and eosinophils in sputum in patients with EIB do not exclude the presence of mast cells.

The examined studies differ on several crucial points: type of sport, methodology, exercise environment and potential exposure to environmental triggers etc. These differences among studies makes it difficult to draw definitive conclusions. However, there are general considerations about the occurrence of neutrophilic airway inflammation among athletes with asthma that could be linked to the appearance of non-Type 2 inflammation in athletes with asthma. The recent studies on mast cells playing a central role by release of key mediators causing airway inflammation and bronchoconstriction needs to be followed up by examinations on elite athletes.

## Pheno- and endotypes in athletes with asthma

Type 2 inflammation in asthma includes inflammatory pathways with Th2- and ILC2 cells which secrete different cytokines e.g., IL-4, IL-5 and IL-13. Moreover, these cells stimulate Th2 immunity, which is characterized by higher levels of immunoglobulin E (IgE), positive skin prick tests (SPT) and eosinophilia (5), whereas ILC2 cells through IL-5 stimulate an eosinophilic inflammation without IgE involvement. The classical Th2-immunity represents the typical adaptive response to allergen exposure in atopic individuals with both IgE and eosinophilic cells. Around 50%–70% of patients with asthma are estimated to have Type 2 asthma and this group typically includes allergic asthma, late-onset eosinophilic asthma together with non-eosinophilic asthma (61, 62). In the past decade, there has been a lot of focus on asthma pheno- and endotypes among non-athletic patients and the immunological mechanisms involved. Researchers have established tremendous knowledge about Type 2 inflammation, but less is known about non-Type 2 inflammation which is present in a subgroup of asthma patients (5). Non-Type 2 asthma is recognized among patients with late-onset of asthma in the grown up, especially women, in the obese, and people who smoke tobacco while asthma among athletes, who do not show sign of classic asthma are not yet established as a non-Type 2 inflammatory disease in the literature (5, 63–65). However, athletes with asthma might also be classified as non-Type 2 asthma, as the majority of studies on airway inflammation in athletes with asthma shows presence of neutrophilia in sputum (36, 44, 45, 47). Results from a recent retrospective, cross-sectional study found that Type 2 asthma is the most prevalent endotype among athletes, however as many as 30% of the athletes have non-Type 2 asthma, which is higher than expected in a population of young athletes (23).

Only a few studies attempt to separate and examine the differences in cell types as well as biomarkers between classic asthma and asthma among elite athletes, in an effort to classify these phenotypes as non-Type 2 asthma or Type 2 asthma. In a study by Couto et al. (31) 150 athletes were divided into groups according to their asthma phenotype (“atopic asthma” vs. “sports asthma”) using latent class analysis. Athletes with “atopic asthma” were characterized by allergic sensitization, rhinitis and other allergic co-morbidities and increased fractional exhaled nitric oxide (FeNO) levels. Athletes with “sports asthma” were characterized by respiratory symptoms during exercise and AHR and absence of allergic conditions. Moreover, exposure to particular environmental conditions of training and competition was associated with increased risk to develop “sports asthma” phenotype: water sports increased the risk by almost three times, whereas in winter sports the risk increased by almost nine times. Tsukioka et al. (66) examined and diagnosed asthma in 104 Japanese athletes and separated these athletes into three clusters based on data from lung function and biomarkers before the induction of therapy. Cluster 1 (32% of athletes) was characterized with moderate levels of FeNO and total IgE. Cluster 2 (44% of athletes) had the lowest levels of FeNO, total IgE concentrations, and peripheral eosinophil counts, lower FEV1 (%) values despite having fewer symptoms. Cluster 3 (24% of athletes) had a history of pediatric asthma and had atopic features, which included higher levels of FeNO, total IgE, and blood eosinophil count, and a greater airway response to methacholine. Cluster 1 and cluster 2 correspond with the “sports asthma” phenotype whereas cluster 3 is comparable with “atopic asthma” described by Couto et al. All athletes received treatment with either ICS alone or an ICS/LABA combination for at least 6 months, based on a physician's judgment. Results found significant decrease in FeNO values in cluster 3. Moreover, significantly improved FEV1 (%) were found in cluster 2 and FEV1 (%) values in subjects treated with an ICS/LABA combination were greatly improved compared to those in subjects treated with an ICS alone which could indicate poorer treatment response with use of only ICS in athletes with asthma who lack the presence of typical atopic parameters.

It is possible that classic Type 2 inflammatory asthma developed in adolescence can coexist with non-Type 2 asthma developed due to sports. This could potentially mix the endotypes (Type 2 / non-Type 2) among the population of athletes with asthma, and there is a risk of misdiagnosing.

## Biomarkers and sputum

In clinic, examination of biomarkers relevant for asthma such as FeNO together with blood eosinophil and total IgE can give an insight into a possible ongoing inflammation and is useful in monitoring of adherence and consideration of medical dose adjustment of ICS (67). Moreover, SPT is used to diagnose IgE-mediated allergic disease e.g., asthma. Measurement of FeNO, blood eosinophils and total IgE has never been done systematically in elite athletes suspected of asthma. At this time, there is no systematic approach to examining various subtypes of asthma including athletes with asthma, however, the use of specific biomarkers, SPT and objective measurements together with detailed description of symptoms and knowledge of competing diseases may provide indications of which subtype could be involved. Non-Type 2 asthma is more difficult to define, as there are no specific biomarkers, others than the absence of typical biomarkers seen in Type 2 asthma.

One way to examine the characteristics of airway inflammation is by analyzing sputum. Sputum from athletes with asthma could contribute with important knowledge to the field because it provides a more accurate insight into different cells as part of the airway inflammation and would illustrate the level and type of airway inflammation from the lower respiratory tract. In addition, sputum analysis could be relevant in interpretation and comparison of inflammation present among athletes and their specific type of sport. But although sputum analysis is relatively simple to perform, it is only done at specific centers, which limits the availability for athletes.

## Treatment

Asthma is the most common chronic disease among Olympics athletes, and it is in the spirit of sports that all athletes should have equal competitive conditions. Therefore, it is important that athletes with asthma receive optimal treatment which enables them to attend to the same extend as healthy individuals and to secure that any long-lasting harm on the airways or disease for the athletes are reduced. Currently, the management of asthma in elite athletes is like asthma in the general population. Standard treatment includes inhaled corticosteroid (ICS) and often daily bronchodilation agents, based on GINA guidelines.

Response to treatment with ICS in patients with asthma seems to depend on the type of asthma and reflect the heterogeneity in asthma. Regarding response to ICS in T2-low and non-Type 2 asthma studies are showing conflicting results (68–70). A recent study by Hvidtfeldt et al. showed improvement in response to the indirect bronchial challenge with mannitol after 1 year of real-life specialist management in patients with both T2-low and T2-high asthma suggesting that patients with T2-low asthma benefits from anti-asthmatic treatment with ICS (71). Even though studies have found beneficial effect of ICS on elite athletes with asthma and EIB (72), there are some indications that the response to ICS in elite athletes with asthma is lower than in the general population (73, 74). A study of Japanese asthmatic athletes found that after at least 12-weeks of intervention 16.3% of the athletes had unsatisfactory response to treatment with ICS. Moreover, results found that athletes less responsive to treatment were characterized by a decreased response to methacholine and lower Th2-associated biomarkers relative to responsive athletes (73). Sue-Chu et al. (74) examined the effect of daily inhaled budesonide for 22-weeks in competitive cross-country skiers with bronchial biopsy and bronchoalveolar lavage (BAL) and found no beneficial effect on AHR to methacholine or changes regarding cellular inflammation in the bronchial mucosa. These results are supporting the non-Type 2 endotype in sports asthma, but more studies on response to treatment in athletes are needed. However, other studies on athletes with asthma finds increased levels of eosinophilic airway inflammation in sputum which significantly attenuated after treatment with high-dose ICS treatment (75). Currently, non-Type 2 asthma is treated the same way as Type 2 asthma with ICS as a fundamental part, but since non-Type 2 asthma contains different immunopathogenesis the response to ICS could be lower. Moreover, patient with non-Type 2 asthma is less responsive to biological treatment which targets Type 2 asthma (76). In athletes, an option to monitor the beneficial effect of ICS treatment could be regular control of indirect AHR testing (77). However, more studies on AHR and monitoring response to treatment in athletes are necessary. In an era of personalized medicine, a better understanding of the underlying endotypes of asthma is essential for future treatment and provide insight into the causes of an unsatisfying response to treatment.

In athletes with asthma who do not respond as expected with reduced respiratory symptoms after initiating standard treatment, alternative treatment must be considered. But since treatment options in elite athletes are limited due to World Anti-Doping Agency (WADA) regulations physicians needs to be aware of available treatment and exclude potential differential diagnosis.

Many elite athletes with asthma use SABA as needed, and with respiratory symptoms in relation to training, athletes are at risk of having high consumption of SABA on a weekly basis (78). This increase the risk of development of tachyphylaxis, which is progressive decrease in response to treatment. Although no RCT studies have been performed in elite athletes, our clinical experience suggests to always treat elite athletes with daily combinational therapy (ICS/LABA) or leukotriene receptor antagonist (LTRA). To protect against tachyphylaxis treatment with beta-2 agonist should be reserved for days with competition and practitioners could consider using long-acting muscarine antagonist (LAMA) daily or two hours before exercise as they do not cause tolerance (79). Moreover, the discovery of the role of mast cells in both Type 2 and non-Type 2 asthma could perhaps be a future asthma treatment option.

## Reversibility of airway hyperresponsiveness

Even though asthma severity is known to vary with age, asthma seldom disappears. Over the past decades, the increased asthma prevalence in elite athletes has been addressed, but research is lacking on how respiratory symptoms, airway hyperresponsiveness and inflammation change after cessation of elite training. No studies have examined whether non-Type 2 asthma in elite athletes might be less chronic and if AHR more frequently disappear. Only few studies have examined how AHR in athletes responds to shorter training breaks or termination of sport. In a study by Bougault et al. nineteen competitive swimmers without a previous diagnosis of asthma performed lung function tests including methacholine challenge test twice over a 1-year period (80). The first test was conducted during an intense period of training. The second test was performed after at least two weeks of absence of training or light-intensity swimming. Observations on methacholine challenge showed that airway hyperresponsiveness was significantly reduced in swimmers after at least two weeks without intense swimming. These findings suggest that AHR to methacholine vary depending on the training intensity (hour per week) and could be considered as a transient condition.

Couto et al. (81) assessed how airway inflammation changes in swimmers during a 3-year follow-up. The study found that those who remained active at follow-up significantly increased their levels of airway inflammation measured by FeNO independently of their gender, age, atopy or asthma status. Helenius et al. (82) have performed a follow-up study of elite swimmers with asthma and found that athletes with asthma who had retired had fewer asthma symptoms and lost their airway hyperresponsiveness to methacholine, and in some athletes asthma had disappeared. These findings indicate that asthma caused by elite training is partly, or maybe even fully, reversible in some athletes. However, more follow-up studies regarding asthma in elite athletes, involving multiple sports, are needed and will contribute with important knowledge in the field.

## Conclusion

Athletes with asthma might be represented by different subtypes of asthma with immunopathological differences. Elite training is suspected to cause asthma but it is to simplified to conclude that elite training on its own is causing asthma in athletes. Concurrent atopy, exposure to enviromental triggers together with training intensity and duration also play a certain role. The exact mechanisms underlying AHR among athletes are incompletely understood. However, the current knowledge on this field leads us to believe that a part of elite athletes with asthma who do not shown signs of Type 2 mediated asthma might be characterized with non-Type 2 asthma. Further studies on active and recreational elite athletes with asthma involving multiple sports, are needed and will contribute with important knowledge in the field of phenotypes and endotype in asthma.

## Author contributions

SMR wrote the manuscript with help from ESHH and VB. All authors contributed to the article and approved the final manuscript.
